# Second-Order Disjoint Factor Analysis

**DOI:** 10.1007/s11336-021-09799-6

**Published:** 2021-08-17

**Authors:** Carlo Cavicchia, Maurizio Vichi

**Affiliations:** 1 Econometric Institute, Erasmus School of Economics, Erasmus University Rotterdam, Rotterdam, The Netherlands; 2 Department of Statistical Sciences, University of Rome La Sapienza, Rome, Italy

**Keywords:** factor analysis, hierarchical models, latent variable models, reflective models, second-order

## Abstract

Hierarchical models are often considered to measure latent concepts defining nested sets of manifest variables. Therefore, by supposing a hierarchical relationship among manifest variables, the general latent concept can be represented by a tree structure where each internal node represents a specific order of abstraction for the latent concept measured. In this paper, we propose a new latent factor model called second-order disjoint factor analysis in order to model an unknown hierarchical structure of the manifest variables with two orders. This is a second-order factor analysis, which—respect to the second-order confirmatory factor analysis—is exploratory, nested and estimated simultaneously by maximum likelihood method. Each subset of manifest variables is modeled to be internally consistent and reliable, that is, manifest variables related to a factor measure “consistently” a unique theoretical construct. This feature implies that manifest variables are positively correlated with the related factor and, therefore, the associated factor loadings are constrained to be nonnegative. A cyclic block coordinate descent algorithm is proposed to maximize the likelihood. We present a simulation study that investigates the ability to get reliable factors. Furthermore, the new model is applied to identify the underlying factors of well-being showing the characteristics of the new methodology. A final discussion completes the paper.

## Introduction

Factor analysis (FA, Anderson & Rubin, 1956; Horst, 1965) is one of the most used models to reconstruct manifest variables (MVs) through a set of latent variables. However, when the studied latent concepts present a hierarchical structure, FA is not an appropriate method because it is not able to model the hierarchical structure of the concepts; therefore, a model with a hierarchical form is required. In psychometric studies, an epitome is the big five model, which is used to measure the big five personality traits of individuals. It is worth mentioning that several studies have stressed the hierarchical relations among such traits running from the more abstract to more specific (Cattell, 1947; de Raad & Mlačić, 1970; Digman, 2015; Eysenck, 1990). Most typically, hierarchies are studied in two ways: either following a bottom-up approach or a top-down approach (Goldberg, 2006). In this paper, we will be focusing on the bottom-up approach, starting from the MVs up to a general factor.

In multidimensional data analysis, two main classes of models were developed to analyze the hierarchical structures of a multidimensional phenomenon: the higher-order factor models (Cattell, 1948; Thompson, 1978) and the hierarchical factor models (Holzinger & Swineford, 1937). On the one hand, higher-order factor analysis provides a different hierarchical prospective of the data (Thompson, 2004), where factors are supposedly correlated. A frequent procedure to obtain higher-order factors consists of the *sequential* application of exploratory factor analysis (EFA) followed by an oblique rotation method. After performing EFA, an oblique rotation method is applied in order to extract the simple structure (Thurstone, 1947) and the first-order factors. This *sequential* application continues to operate on the correlation matrix of the factors from the first-order upwards, until a single factor or an uncorrelated set of factors are identified (Gorsuch, 1983). Although the oblique rotation after EFA is used to identify the simple structure, Vichi (2017) showed that this sequential approach may fail to find the simple structure, hence underlying that a more specific methodology is necessary.

On the other hand, hierarchical factor models are characterized by a single order of orthogonal hierarchical factors usually obtained by applying the Schmid–Leiman transformation (Schmid & Leiman, 1957) to the corresponding higher-order solutions. Therefore, higher-order models identify the effect of the general factor on MVs only through the higher-order factors, whereas the hierarchical models also identify a direct effect of the general factor on the MVs. However, the link between the higher-order and hierarchical factor models was eventually established by Yung et al. (1999) determining the conditions for their equivalence.

It is worth remarking that many hierarchical extensions of FA were already proposed throughout the years (Le Dien & Pages, 2003; Schmid & Leiman, 1957; Thompson, 1951; Wherry, 1959; 1975; 1984). However, all of these hierarchical extensions were developed as sequential analysis, at times not even guaranteeing to obtain a simple structure, i.e., the partition in *H* classes of variables where common relations in each class are represented by a single factor.

A hierarchical model which may consider a simple structure of the MVs is the *Hierarchical Confirmatory Factor Analysis* (HCFA, Holzinger, 1944; Jöreskog, 1966; 1969; 1978; 1979). This latter is often used to measure general latent concepts when the number of factors and the most relevant relations between MVs and factors are known. However, such knowledge may also represent a limitation. Firstly, because the researcher might not have the a priori information concerning the relations between the different levels of factors and between variables and factors, or, secondly, because the theory might turn out to be erroneous in some parts or at least in its empirical application.

The researcher can overcome these issues accepting that each variable is related with a latent construct only, without imposing what the relations between MVs and factors are. The result is to obtain an exploratory simple structure model (SSM) where the relations between MVs and factors are determined by the data. It is worth recalling that many researchers have investigated factorial methods to obtain a simple structure. For instance, Hirose and Yamamoto (2014) developed a FA with non-convex sparse penalty which can provide a simple structure. Vichi (2017) proposed a model, named disjoint factor analysis (DFA), to identify the best SSM for the data, wherein the maximum likelihood estimation allows to make inference on the number of factors, on the relations between MVs and factors (i.e., loadings), and to assess the validity of the SSM for the observed data. Adachi and Trendafilov (2018) also proposed a matrix-based procedure for sparse FA such that each variable loads only on one common factor by obtaining a simple structure.

In this paper, we extend DFA, which is not appropriate to identify the hierarchical structure of factors since it assumes that factors are orthogonal, that is, factors are not mutually related and do not share common information that could be summarized by the general factor. Therefore, we release the orthogonal constraint and assume that the correlation structure in the data has an unknown second-order hierarchical form, where the first order indicates a reduced set of multidimensional concepts described by disjoint subsets of MVs, while the second order, denoted root or general level, represents the general factor. Gorsuch (1983) emphasized that the interpretation of higher-order factor models should be based on the MVs, in order to avoid interpretations of interpretations; this can be certainly achieved when disjoint classes of variables can be identified with each class represented by a factor.

**Fig. 1:**
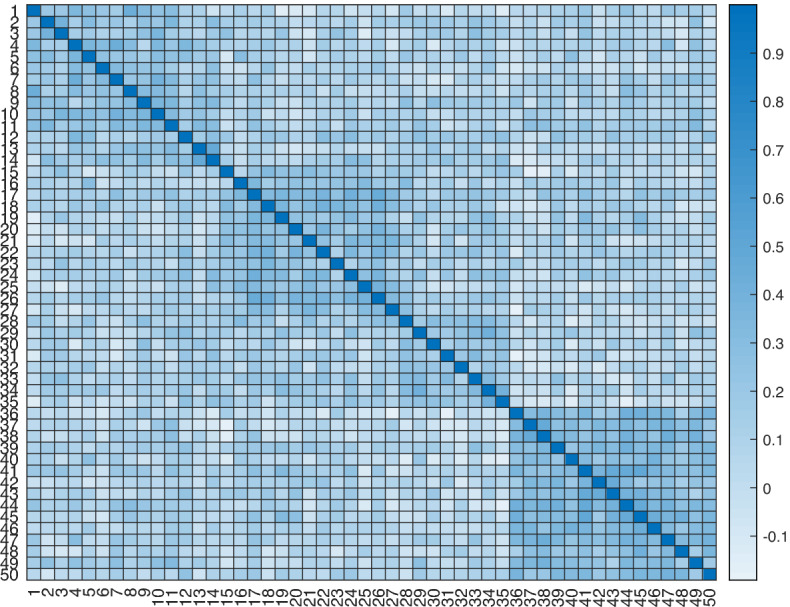
($50 \times 50$) Correlation matrix with a block diagonal structure in four blocks

Finally, it is crucial to remark that the *sequential* application of the EFA followed by an oblique rotation method cannot efficiently identify the block correlation structure, whereas a simultaneous and exploratory model can. To verify this, we generated a dataset of 500 observations according to the block diagonal model 16 corresponding to second-order factor analysis. Correlations within blocks are on average around 0.85, while correlations between blocks are around 0.35 (e.g., the heatmap in Fig. 1). Four blocks of variables were considered: ($V_1 - V_{14}$), ($V_{15} - V_{27}$), ($V_{28} - V_{35}$) and ($V_{36} - V_{50}$). Each variable was assigned to the factor with the highest loading resulting from the *sequential* application of the EFA followed by an oblique rotation method. To assess the similarities between two partitions, induced by disjoint blocks of variables, we computed the adjusted Rand index (ARI, Hubert & Arabie, 1985) between the generated blocks and those identified by the methodology. The ARI is the corrected-for-chance version of the Rand index (Rand, 1971). This index has zero expected value in the case the identified partition is a random one, and it is bounded above by 1 in the case of a perfect agreement between the identified and the generated partitions. The ARI between the partition found by EFA followed by an oblique rotation method and the generated one resulted equal to 0.78; our methodology, applied to the same dataset, was able to perfectly detect the generated blocks (i.e., ARI $=1$). To extend this result to a reasonable number of examples, we generated 200 samples with the same four-block structure. The ARI between the EFA followed by an oblique rotation method and the generated data resulted equal to 1 for 115 times ($57,5\%$), whereas our methodology perfectly detected the generated structure in 144 cases ($72\%$). So far, our proposed model detected the true structure in sensibly more times than EFA followed by an oblique rotation method, and the averages of ARI resulted equal to 0.89 for our methodology and 0.45 for EFA followed by an oblique rotation method.

The paper is organized as follows. In Sect. 2, we propose the Second-Order Factor Analysis model. Section 3 includes an overview of disjoint models. Section 4 introduces the nonnegative constraints of the factors necessary to specify consistent latent variables. A simulation study is considered in Sect. 5. Section 6 shows an application about well-being. A final discussion completes the paper in Sect. 7.

## Second-order factor analysis

Let $\mathbf {x}$ be the ($J \times 1$) multivariate random variable with mean vector ${\varvec{\mu }}_\mathbf {x} = [{\varvec{\mu }}_1,\dots ,{\varvec{\mu }}_J]' = \mathbf {0}_J$ without loss of generality, and *J*-dimensional variance–covariance matrix ${\varvec{\Sigma }}_\mathbf {x}$. Second-Order Factor Analysis (2O-FA) is modeled as a new factor model that considers two typologies of latent unknown constructs: ($H \le J$) first-order factors and a single (nested) general factor identified by the two simultaneous equations

1 $$\begin{aligned} \mathbf {x}&= \mathbf {Ay}+\mathbf {w} \end{aligned}$$

2 $$\begin{aligned} \mathbf {y}&= \mathbf {c}g+\mathbf {u} \end{aligned}$$

where $\mathbf {A}$ is the ($J \times H$) matrix of unknown first-order factors loadings, $\mathbf {y}$ is the non-observable ($H \times 1$) random vector denoting the first-order factor scores, and $\mathbf {w}$ is a non-observable ($J \times 1$) random vector of errors. It is assumed that $\mathbf {y} \sim N_H(\mathbf {0},{\varvec{\Sigma }}_\mathbf {y})$, where ${\varvec{\Sigma }}_\mathbf {y}$ is the correlation matrix of the first-order factors, and $\mathbf {w} \sim N_J(\mathbf {0},{\varvec{\Psi }}_\mathbf {x})$, where $\mathrm {cov}(\mathbf {w})={\varvec{\Psi }}_\mathbf {x}$ is the *J*-dimensional diagonal positive definite variance–covariance matrix of the error of model (1) and $\mathrm {cov}(\mathbf {w},\mathbf {y})=\mathbf {0}$.

Furthermore, *g* is a non-observable random variable normally distributed with mean 0 and variance $\mathrm {cov}(g)=1$, and $\mathbf {c}$ is the ($H \times 1$) vector of unknown general factor loadings. In addition, $\mathbf {u}$ is a non-observable ($H \times 1$) random vector of errors. It is assumed that $\mathbf {u} \sim N_H (\mathbf {0},{\varvec{\Psi }}_\mathbf {y})$, where $\mathrm {cov}(\mathbf {u})={\varvec{\Psi }}_\mathbf {y}$ is the *H*-dimensional diagonal positive definite variance–covariance matrix of the error of model (2). In addition, it is assumed that errors in the two models are uncorrelated $\mathrm {cov}(\mathbf {w},\mathbf {u})=\mathbf {0}$; and errors and factors are uncorrelated, i.e., $\mathrm {cov}(\mathbf {u},g)=\mathbf {0}$.

Model (1) identifies *H* specific theoretical constructs by means of a common factor model that identifies common information with *H* factors related to the MVs, while model (2) detects the general latent construct by means of a one-factor model that identifies common information with one general factor related to the *H* first-order factors.

Given these assumptions and including (2) into (1), the 2O-FA model for centered data is defined

3 $$\begin{aligned} \mathbf {x} = \mathbf {A}(\mathbf {c}g+\mathbf {u})+\mathbf {w} \text {.} \end{aligned}$$

It can be derived that $\mathbf {x} \sim N_J (\mathbf {0}_J,{\varvec{\Sigma }}_\mathbf {x})$, where the variance–covariance matrix ${\varvec{\Sigma }}_\mathbf {x}$ is

4 $$\begin{aligned} {\varvec{\Sigma }}_\mathbf {x}&= \mathrm {cov}(\mathbf {A}\mathbf {c}g + \mathbf {A}\mathbf {u}+\mathbf {w}) \nonumber \\&= \mathbf {A}\mathbf {c}\;\mathrm {cov}(g)\mathbf {c}'\mathbf {A}' + \mathbf {A}\;\mathrm {cov}(\mathbf {u})\mathbf {A}'+\mathrm {cov}(\mathbf {w}) \nonumber \\&=\mathbf {A}\mathbf {c}\mathbf {c}'\mathbf {A}'+\mathbf {A}{\varvec{\Psi }}_\mathbf {y}\mathbf {A}'+{\varvec{\Psi }}_\mathbf {x} \nonumber \\&= \mathbf {A}{\varvec{\Sigma }}_\mathbf {y}\mathbf {A}'+{\varvec{\Psi }}_\mathbf {x} \end{aligned}$$

with

5 $$\begin{aligned} {\varvec{\Sigma }}_\mathbf {y} = \mathbf {c}\mathbf {c'}+{\varvec{\Psi }}_\mathbf {y}. \end{aligned}$$

Matrix ${\varvec{\Sigma }}_\mathbf {y}$ is a correlation matrix since first-order factors are standardized. Similarly to exploratory factor analysis, 2O-FA (3) does not imply any a priori knowledge of relations between MVs and factors. Although many hierarchical extensions of FA are present into specialized literature (e.g., Le Dien & Pages, 2003; Schmid & Leiman, 1957; Thompson 1951; Wherry, 1959; 1975; 1984), the novelty of the proposal consists of the covariance structure given by the model (4) and (5) and the simultaneous estimation of the parameters.

Let us consider a random sample of $n > J$ multivariate observations $\mathbf {x}_i=[x_{i1},\dots ,x_{iJ}]'$, $i=1,\dots ,n$ drawn of $\mathbf {x}$, with mean vector $\bar{\mathbf {x}}$, and *J*-dimensional variance–covariance matrix $\mathbf {S}_{\mathbf {x}}= \frac{1}{n} \sum _{i=1}^n \mathbf {x}_i\mathbf {x}_i'$, the model (3) in matrix form corresponds to

6 $$\begin{aligned} \mathbf {X}=\mathbf {gc}'\mathbf {A}'+ \mathbf {E} \end{aligned}$$

where $\mathbf {X} = [\mathbf {x}_1,\dots ,\mathbf {x}_{n}]'$ is the $(n \times J)$ matrix containing the *n* multivariate observations, $\mathbf {g} = [g_1,\dots ,g_{n}]'$ is the non-observable $(n \times 1)$ vector denoting the second-order (general) factor scores and $\mathbf {E}=\mathbf {UA}'+\mathbf {W}$. In detail, $\mathbf {W}=[\mathbf {w}_1,\dots ,\mathbf {w}_{n}]'$ with dimensions $(n \times J)$ and $\mathbf {U}=[\mathbf {u}_1,\dots ,\mathbf {u}_{n}]'$ with dimensions $(n \times H)$ are matrices containing the non-observable errors related to (1) and (2), respectively. The reduced log-likelihood (i.e., conditional on ${\varvec{\mu }}_{\mathbf {x}}$ equal to the sample mean) is

7 $$\begin{aligned} L( \mathbf {A}, \mathbf {c}, {\varvec{\Psi }}_\mathbf {x}, {\varvec{\Psi }}_\mathbf {y})= & {} -\frac{nJ}{2}\log (2\pi )-\frac{n}{2}\{\log |\mathbf {A}(\mathbf {c}\mathbf {c}'+ {\varvec{\Psi }}_\mathbf {y})\mathbf {A}'+{\varvec{\Psi }}_\mathbf {x}|\nonumber \\&+\,\mathrm {Tr}[(\mathbf {A}(\mathbf {c}\mathbf {c}'+ {\varvec{\Psi }}_\mathbf {y})\mathbf {A}'+{\varvec{\Psi }}_\mathbf {x})^{-1}\mathbf {S}_{\mathbf {x}}]\}\text {.} \end{aligned}$$

## Disjoint Models

Disjoint orthogonal factor analysis (DFA, Vichi, 2017) assumes that observations can be reconstructed by a non-observable ($H \times 1$) random vector $\mathbf {y}$ denoting a reduced set of ($H \le J$) common factors. DFA for centered data can be expressed via the following model

8 $$\begin{aligned} \mathbf {x}=\mathbf {BV}\mathbf {y}+\mathbf {w} \end{aligned}$$

with a covariance structure equal to (4), considering the loading matrix $\mathbf {A}$ restricted to the product

9 $$\begin{aligned} \mathbf {A}=\mathbf {B}\mathbf {V} \end{aligned}$$

where $\mathbf {V}=[v_{jh}]$ is a ($J \times H$) binary and row stochastic matrix identifying a partition of MVs into *H* subsets corresponding to *H* factors; the *H* subsets of MVs are denoted as $C_h$, with $h=1, \dots , H$. If the *j*th MV belongs to the *h*th subset then $v_{jh}=1$, otherwise, $v_{jh}=0$; whereas, $\mathbf {B}=\mathrm {diag}(b_1,\dots ,b_J)$ is a ($J \times J$) weighting diagonal matrix such that $b_j^2 > 0$. If it is allowed $b_j^2 \ge 0$, when $b_j^2 = 0$ the DFA admits a model selection feature, that is, a MV is assigned to a subset with a loading equal to zero. In this case, the MV *j* is discarded from the model. Despite the fact that DFA assumes orthogonal factors, that is, ${\varvec{\Sigma }}_{\mathbf {y}}=\mathbf {I}_H$, in this paper this condition is relaxed in order to allow a hierarchical structure of the data. Note that $\mathrm {diag}(\cdot )$ produces a diagonal matrix of a vector.

Therefore, formally, DFA corresponds to (1) with covariance structure given by (4) once the loading matrix $\mathbf {A}$ is defined according to (9). The model is defined under the following constraints

10 $$\begin{aligned}&\mathbf {V} = [v_{jh} \in \{0,1\}:j = 1,\dots ,J, h = 1,\dots ,H] \end{aligned}$$

11 $$\begin{aligned}&\mathbf {V}\mathbf {1}_H = \mathbf {1}_J \quad \text {i.e.} \quad \sum _{h = 1}^{H} v_{jh} = 1 \quad j = 1,\dots ,J \end{aligned}$$

12 $$\begin{aligned}&\mathbf {B}=\mathrm {diag}(b_1,\dots ,b_J) \end{aligned}$$

13 $$\begin{aligned}&\mathbf {V}'\mathbf {BBV}=\mathrm {diag}(b_{\cdot 1}^2,\dots ,b_{\cdot H}^2) \quad \text {with} \quad b_{\cdot h}^2 = \sum _{j \in C_h} b_{j}^2. \end{aligned}$$

In detail, if ${\varvec{\Sigma }}_{\mathbf {y}}=\mathbf {I}_H$ (disjoint orthogonal factor analysis) the variance–covariance matrix ${\varvec{\Sigma }}_{\mathbf {x}}$ is block diagonal:

14 $$\begin{aligned} {\varvec{\Sigma }}_{\mathbf {x}}=\mathrm {blkdiag}({\varvec{\Sigma }}_{11},\dots ,{\varvec{\Sigma }}_{hh},\dots ,{\varvec{\Sigma }}_{HH})= \begin{bmatrix} {\varvec{\Sigma }}_{11} &{} 0 &{} \dots &{} \dots &{} \dots &{} 0\\ 0 &{} {\varvec{\Sigma }}_{22} &{} 0 &{} \dots &{} \dots &{} 0\\ \vdots &{} 0 &{} \ddots &{} 0 &{} \dots &{} 0\\ \vdots &{} \vdots &{} 0 &{} {\varvec{\Sigma }}_{hh}&{} 0 &{} 0\\ \vdots &{} \vdots &{} \vdots &{} 0 &{} \ddots &{} 0\\ 0 &{} 0 &{} 0 &{} 0 &{} 0 &{} {\varvec{\Sigma }}_{HH} \\ \end{bmatrix}\text {.} \end{aligned}$$

Each block is the variance–covariance matrix ${\varvec{\Sigma }}_{hh}$ of the MVs relating to the factor *h*,

15 $$\begin{aligned} {\varvec{\Sigma }}_{hh}=\mathbf {B}_h(\mathbf {1}_{n_h}\mathbf {1}_{n_h}')\mathbf {B}_h+{\varvec{\Psi }}_h=\mathbf {b}_h\mathbf {b}'_h+{\varvec{\Psi }}_h \end{aligned}$$

where $\mathbf {B}_h=\mathrm {diag}(\mathbf {b}_h)$, $\mathbf {b}_h=[b_{1h},\dots ,b_{n_hh}]'$ and ${\varvec{\Psi }}_h=\mathrm {diag}({\varvec{\Psi }}_h)$, ${\varvec{\Psi }}_h=[{\varvec{\Psi }}_{1h},\dots ,{\varvec{\Psi }}_{n_hh}]'$. Note that $n_h$ is the number of MVs related to the latent factor *h*. Therefore, DFA assumes that a relevant correlation among MVs related to the same latent factor is observed, while a negligible correlation between MVs related to different latent factors is detected.

If ${\varvec{\Sigma }}_{\mathbf {y}}$ has non-diagonal elements different from zero (disjoint non-orthogonal factor analysis), the block diagonal variance–covariance disappears and ${\varvec{\Sigma }}_{\mathbf {x}}$ has the form

16 $$\begin{aligned} {\varvec{\Sigma }}_{\mathbf {x}}= \begin{bmatrix} {\varvec{\Sigma }}_{11} &{} {\varvec{\Sigma }}_{12} &{} \dots &{} {\varvec{\Sigma }}_{1H}\\ {\varvec{\Sigma }}_{21} &{} {\varvec{\Sigma }}_{22} &{} \dots &{} {\varvec{\Sigma }}_{2H}\\ \vdots &{} \vdots &{} {\varvec{\Sigma }}_{hh} &{} \vdots \\ {\varvec{\Sigma }}_{H1} &{} {\varvec{\Sigma }}_{H2} &{} \dots &{} {\varvec{\Sigma }}_{HH} \\ \end{bmatrix} \end{aligned}$$

where the generic matrix correlation between two subsets of MVs is constrained in

17 $$\begin{aligned} {\varvec{\Sigma }}_{hk} = c_h c_k\mathbf {B}_h(\mathbf {1}_{n_h}\mathbf {1}_{n_k}')\mathbf {B}_k=c_h c_k\mathbf {b}_h\mathbf {b}'_k \end{aligned}$$

and $c_h c_k$ expresses the correlation between factor *h* and factor *k*.

It is worth noticing that in order to identify two distinct factors *h* and *k* with two associated disjoint subsets of MVs defining matrices ${\varvec{\Sigma }}_{hh}$ and ${\varvec{\Sigma }}_{kk}$, both a high correlation within these matrices and a lower (or equal to, if and only if $c_h$ and $c_k$ are both equal to 1) correlation within ${\varvec{\Sigma }}_{hk}$ need to be observed. In fact, if a high correlation is also observed in ${\varvec{\Sigma }}_{hk}$, then a single factor in the data is present, since the two subsets of MVs are not distinct and actually form a single subset in which MVs are all highly correlated. Thus, (17) guarantees that correlations in ${\varvec{\Sigma }}_{hk}$ are lower than correlations within ${\varvec{\Sigma }}_{hh}$ and ${\varvec{\Sigma }}_{kk}$.

###  Second-Order Disjoint Factor Analysis

**Fig. 2:**
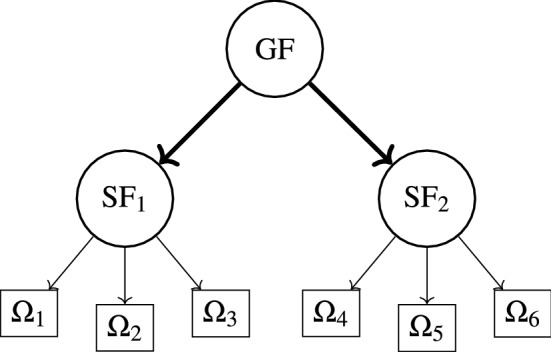
Example of second-order disjoint factor model

When in the 2O-FA some a priori substantial knowledge is incorporated in the form of restrictions on the loading matrix, this usually improves the description of the latent factors and leads to a parsimonious model with a simple loading matrix structure. Therefore, if in 2O-FA a SSM is assumed observed for the data, this means that the factor loading matrix has the form $\mathbf {A}=\mathbf {BV}$ (Eq. 9) and 2O-FA becomes *Second-Order Disjoint Factor Analysis* (2O-DFA, Fig. 2).

Once the loading matrix $\mathbf {A}$ is defined according to (9), 2O-DFA is defined by (3), or alternatively in matrix form by (6). It is worth observing that the maximization of (7) corresponds to the minimization of

18 $$\begin{aligned} D(\mathbf {B},\mathbf {V}, \mathbf {c}, {\varvec{\Psi }}_\mathbf {x}, {\varvec{\Psi }}_\mathbf {y}) = \log |\mathbf {BV}(\mathbf {c}\mathbf {c}'+{\varvec{\Psi }}_{\mathbf {y}})\mathbf {V}'\mathbf {B}+{\varvec{\Psi }}_{\mathbf {x}}|+\mathrm {Tr}\{[\mathbf {BV}(\mathbf {c}\mathbf {c}'+{\varvec{\Psi }}_{\mathbf {y}})\mathbf {V}'\mathbf {B}+{\varvec{\Psi }}_{\mathbf {x}}]^{-1}\mathbf {S}_{\mathbf {x}}\}\text {.} \end{aligned}$$

### Second-Order Disjoint FA algorithm

Given *H*, a cyclic block coordinate descent algorithm for the estimation of the model can be described by five steps which are sequentially repeated until a stopping rule is satisfied.

**Step 0**: [*Initialization*] A random partition $\widehat{\mathbf {V}}$ is generated from a multinomial distribution in *H* categories each with equal probability, where categories are not empty. Matrices $\widehat{{\varvec{\Psi }}}_{\mathbf {x}}=\mathrm {diag}(\mathbf {S}_{\mathbf {x}})$, $\widehat{{\varvec{\Psi }}}_{\mathbf {y}}= \mathrm {diag}(\widehat{\psi }_{\mathbf {y}1},\dots ,\widehat{\psi }_{\mathbf {y}H})$, where each value $\widehat{\psi }_{\mathbf {y}h}$, $h=1,\dots ,H$ is generated from an uniform distribution *U*(0, 1), and vector $\widehat{\mathbf {c}}=[{\widehat{c}}_1, \dots , {\widehat{c}}_H]'$, where each value ${\widehat{c}}_h$, $h=1,\dots ,H$ is generated from an uniform distribution *U*(0, 1).
**Step 1**: Given $\widehat{\mathbf {V}}=[\widehat{\mathbf {v}}_{\cdot 1},\dots ,\widehat{\mathbf {v}}_{\cdot H}]$, $\widehat{\mathbf {c}}$, $\widehat{{\varvec{\Psi }}}_{\mathbf {x}}$ and $\widehat{{\varvec{\Psi }}}_{\mathbf {y}}$, the discrepancy function $D(\mathbf {B},\widehat{\mathbf {V}},\widehat{\mathbf {c}},\widehat{{\varvec{\Psi }}}_{\mathbf {x}},\widehat{{\varvec{\Psi }}}_{\mathbf {y}})$ (18) is minimized with respect to $\mathbf {B}_h=\mathrm {diag}(\mathbf {b}_h)$ by
  19 $$\begin{aligned} \widehat{\mathbf {b}}_h = \widehat{{\varvec{\Psi }}}_{\mathbf {x}h}^{\frac{1}{2}}\mathbf {m}_{1h}(\lambda _{1h}-1)^{\frac{1}{2}}\;\;h=1,\dots ,H \end{aligned}$$
  where $\mathbf {S}_{\mathbf {x}h}$ and $\widehat{{\varvec{\Psi }}}_{\mathbf {x}h}$ are the variance–covariance matrix and the current estimation of the diagonal error variance–covariance matrix of MVs identified by $\widehat{\mathbf {v}}_{\cdot h}$, respectively. $\lambda _{1h}$ is the largest eigenvalue of $\widehat{{\varvec{\Psi }}}_{\mathbf {x}h}^{-\frac{1}{2}}\mathbf {S}_{\mathbf {x}h}\widehat{{\varvec{\Psi }}}_{\mathbf {x}h}^{-\frac{1}{2}}$ and $\mathbf {m}_{1h}$ is the associated eigenvector.
**Step 2**: Given $\widehat{\mathbf {A}}=\widehat{\mathbf {B}}\widehat{\mathbf {V}}$, $\widehat{\mathbf {c}}$ and $\widehat{{\varvec{\Psi }}}_{\mathbf {y}}$ the minimization of the discrepancy function (18) with respect to ${\varvec{\Psi }}_{\mathbf {x}}$ is given by
  20 $$\begin{aligned} \widehat{{\varvec{\Psi }}}_{\mathbf {x}} = \mathrm {diag}(\mathbf {S}_{\mathbf {x}}-\widehat{\mathbf {A}}(\widehat{\mathbf {c}}\widehat{\mathbf {c}}'+\widehat{{\varvec{\Psi }}}_{\mathbf {y}})\widehat{\mathbf {A}}')\text {.} \end{aligned}$$
**Step 3**: Given $\widehat{\mathbf {A}}=\widehat{\mathbf {B}}\widehat{\mathbf {V}}$, $\widehat{{\varvec{\Psi }}}_{\mathbf {x}}$ and $\widehat{{\varvec{\Psi }}}_{\mathbf {y}}$ the minimization of the discrepancy function (18) with respect to $\mathbf {c}$ is given by
  21 $$\begin{aligned} \widehat{\mathbf {c}}=\widehat{{\varvec{\Psi }}}_{\mathbf {y}}^{\frac{1}{2}}\mathbf {p}_1(q_1-1)^{\frac{1}{2}} \end{aligned}$$
  where $q_1$ is the largest eigenvalue and $\mathbf {p}_1$ is the associated eigenvector of $\widehat{{\varvec{\Psi }}}_{\mathbf {y}}^{-\frac{1}{2}}(\widehat{\mathbf {A}}^+(\mathbf {S}_{\mathbf {x}}-\widehat{{\varvec{\Psi }}}_{\mathbf {x}})\widehat{\mathbf {A}}'^+)\widehat{{\varvec{\Psi }}}_{\mathbf {y}}^{-\frac{1}{2}}$, and $\mathbf {A}^{+}$ denotes the Moore–Penrose inverse of a matrix $\mathbf {A}$ (i.e., $\mathbf {A}^+=(\mathbf {A}'\mathbf {A})^{-1}\mathbf {A}'$).
**Step 4**: Given $\widehat{\mathbf {A}}=\widehat{\mathbf {B}}\widehat{\mathbf {V}}$, $\widehat{\mathbf {c}}$ and $\widehat{{\varvec{\Psi }}}_{\mathbf {x}}$ the minimization of the discrepancy function (18) with respect to ${\varvec{\Psi }}_{\mathbf {y}}$ is given by
  22 $$\begin{aligned} \widehat{{\varvec{\Psi }}}_{\mathbf {y}} = \mathrm {diag}(\mathbf {I}_H-\widehat{\mathbf {c}}\widehat{\mathbf {c}}') \end{aligned}$$
  where $\mathbf {I}_H$ is the identity matrix of order *H*.
**Step 5**: Partition $\widehat{\mathbf {V}}=[\widehat{\mathbf {v}}_{\cdot 1},\dots ,\widehat{\mathbf {v}}_{\cdot H}]$ is obtained row by row by assigning each *j*th MV to the *h*th subset that most decreases the discrepancy function (18). Thus, formally
  23 $$\begin{aligned} {\left\{ \begin{array}{ll} {\widehat{v}}_{jh} = 1 \quad \text {if} \quad h = \underset{{h' = 1,\dots ,H}}{\mathrm {argmin}}\, D(\widehat{\mathbf {B}},[\widehat{\mathbf {v}}_{1\cdot },\dots ,\widehat{\mathbf {v}}_{j\cdot } = \mathbf {i}_{h'\cdot },\dots ,\widehat{\mathbf {v}}_{J\cdot }]', \widehat{\mathbf {c} },\widehat{{\varvec{\Psi }}}_\mathbf {x},\widehat{{\varvec{\Psi }}}_\mathbf {y}) \\ {\widehat{v}}_{jh} = 0 \quad \text {otherwise} \end{array}\right. } \end{aligned}$$
  where $\mathbf {i}_{h \cdot }$ is the *h*th column of the identity matrix of order *H*. Note that the update of each row of $\mathbf {V}$ induces the update of the loadings of the two subsets of MVs that are eventually changed. This means that the *j*th MV is assigned to the *h*th subset if Eq. (18) is maximized by ${\widehat{v}}_{jh} = 1$. This step considers a constraint of non-emptiness for each column: thus, the constraints guarantee that each column of $\widehat{\mathbf {V}}$ is not empty and the solution is feasible. In practice, the assignment of a MV is not allowed if this makes a column of $\widehat{\mathbf {V}}$ empty.

In order to better follow the different iterations, the algorithm is presented as follows:

**Step 0**: $\mathbf {V}^{(0)}$, ${\varvec{\Psi }}^{(0)}_{\mathbf {x}}$ and ${\varvec{\Psi }}^{(0)}_{\mathbf {y}}$ randomly generated as described in the initialization step.

For $t=0,\dots ,T-1$, where *T* is the maximum number of iterations:

**Step 1**: $\mathbf {B}^{(t+1)}$ as in the maximum likelihood FA, according to Eq. (19); and $\mathbf {A}^{(t+1)}=\mathbf {B}^{(t+1)}\mathbf {V}^{(t)}$.
**Step 2**: ${\varvec{\Psi }}^{(t+1)}_{\mathbf {x}}$ as we obtain the unique variances, according to Eq. (20).
**Step 3**: $\mathbf {c}^{(t+1)}$ as in the maximum likelihood FA, according to (21).
**Step 4**: ${\varvec{\Psi }}^{(t+1)}_{\mathbf {y}}$ as we obtain the unique variances, according to Eq. (22).
**Step 5**: $\mathbf {V}^{(t+1)}$ according to Eq. (23).

**Step 1**, **2**, **3**, **4** and **5** are therefore alternated repeatedly, and they form a coordinate descent algorithm where, at each iteration (*t*), the discrepancy function decreases or, at least, it does not increase (Zangwill, 1969). The algorithm continues to iterate until the reduction of the discrepancy is larger than an arbitrary small positive constant and $t<T$ (in our experiments *T* was set equal to 100), otherwise the algorithm stops and is considered to have converged to a solution that is not guaranteed to be a global minimum. To avoid the well-known sensitivity of the coordinate descent algorithms to the starting values and to increase the chance of finding the global minimum, the algorithm should be run several times starting from different initial estimates of $\mathbf {V}$ and retaining the best solution. The algorithm generally stops after a few iterations (in our experiments and simulation study less than 15). In order to assess the complexity of 2O-DFA, it is worth recalling that the variance–covariance matrix ${\varvec{\Sigma }}_\mathbf {x}$ has $\frac{J(J+1)}{2}$ elements to be estimated. 2O-DFA reconstructs this matrix in terms of $2J+H$ unknown free parameters in $\mathbf {A}=\mathbf {BV}$, ${\varvec{\Psi }}_\mathbf {x}$ and $\mathbf {c}$. In detail, we have to consider *JH* parameters in $\mathbf {A}$, *J* parameters in ${\varvec{\Psi }}_\mathbf {x}$ and $\frac{H(H-1)}{2}$ parameters in ${\varvec{\Sigma }}_\mathbf {y}$, which is in turn estimated in terms of *H* parameters in $\mathbf {c}$. Yet, in order to obtain a SSM, it is necessary to include $J(H-1)$ constraints in $\mathbf {A}$ (i.e., only one loading per row different from zero). Finally, the effective number of unknown free parameters in 2O-DFA is obtained, and the degrees of freedom are

24 $$\begin{aligned} \nu = \frac{J(J+1)}{2}-JH-H-J+J(H-1) = \frac{J(J+1)}{2} - (2J+H) \text {.} \end{aligned}$$

There are many methods to estimate the factor scores that were proposed throughout the years. For instance, we consider the weighted least square estimation of $\mathbb {E}(\mathbf {X}|\mathbf {Y})$

25 $$\begin{aligned} \widehat{\mathbf {Y}}=\mathbf {X}\widehat{{\varvec{\Psi }}}_{\mathbf {x}}^{-1} \widehat{\mathbf {A}}(\widehat{\mathbf {A}}'\widehat{{\varvec{\Psi }}}_{\mathbf {x}}^{-1}\widehat{\mathbf {A}})^{-1} \end{aligned}$$

as proposed by Bartlett (1937), or simply the Thompson (1934) regression estimator

26 $$\begin{aligned} \widehat{\mathbf {Y}}=\mathbf {X}(\widehat{{\varvec{\Sigma }}}_{\mathbf {x}}^{-1}\widehat{\mathbf {A}})\text {.} \end{aligned}$$

## Second-Order Nonnegative Disjoint Factor Analysis

2O-DFA can be considered to build statistically estimated (i.e., nonnormative) composite indicators (CIs, OECD, 2008) for multidimensional phenomena, when the first-order factors represent specific CIs (one per subset of MVs) and the second-order factor is the general CI. This approach guarantees to comply with certain good properties on which a CI should be based (e.g., model-based with a two-order structure, scale-invariance, uni-dimensionality and reliability). However, the presence of negative loadings can be a limitation since positive relationships among MVs might be compensated by negative ones. First-order loadings show the importance of each MV into the definition of the related first-order factor, while second-order loadings represent the importance of the first-order factors into the definition of the second-order factor; thus, the researcher may constrain the loadings to be positive and consistent. If some or all MVs increase, then also the corresponding first-order factor consistently increases. The same holds for the relationship between the first-order factors and the second-order factor. In order to ensure that loadings remain a measure of importance, the aggregation method should not allow compensability (Greco et al., 2019; Munda & Nardo, 2009; OECD, 2008). We therefore propose a constrained version of 2O-DFA called Second-Order Nonnegative Disjoint Factor Analysis (2ON-DFA).

Let us recall that the discrepancy function (18) is minimized with respect to $\mathbf {B}_h=\mathrm {diag}(\mathbf {b}_h)$ by (19), and that $\lambda _{1h}$ and $\mathbf {m}_{1h}$ minimize $||\widehat{{\varvec{\Psi }}}_{\mathbf {x}h}^{-\frac{1}{2}}\mathbf {S}_{\mathbf {x}}\widehat{{\varvec{\Psi }}}_{\mathbf {x}h}^{-\frac{1}{2}}-\lambda _{1h}\mathbf {m}_{1h}\mathbf {m}_{1h}'||^2$, or equivalently

27 $$\begin{aligned} ||\mathbf {X}_{h}\widehat{{\varvec{\Psi }}}_{\mathbf {x}h}^{-\frac{1}{2}}-\sqrt{\lambda _{1h}}\mathbf {y}_{h}\mathbf {m}_{1h}'||^2 \end{aligned}$$

where $\mathbf {X}_h$ is the data matrix formed by MVs identified by $\mathbf {v}_{\cdot h}$, and $\mathbf {y}_h$ is the factor score vector. The problem in (27) can be solved by an alternating least-squares algorithm that alternates the solution of two regression problems. Given $\widehat{\mathbf {m}}_{1h}$, compute $\widehat{\mathbf {y}}_h$ by

28 $$\begin{aligned} \widehat{\mathbf {y}}_h = \mathbf {X}_h \widehat{{\varvec{\Psi }}}_{\mathbf {x}h}^{-\frac{1}{2}}\widehat{\mathbf {m}}_{1h}(\widehat{\mathbf {m}}'_{1h}\widehat{\mathbf {m}}_{1h})^{-1}\text {.} \end{aligned}$$

Given $\widehat{\mathbf {y}}_h$, compute $\widehat{\mathbf {m}}_{1h}$ by

29 $$\begin{aligned} \widehat{\mathbf {m}}_{1h} = \widehat{{\varvec{\Psi }}}_{\mathbf {x}h}^{-\frac{1}{2}}\mathbf {x}'_h\widehat{\mathbf {y}}_h(\widehat{\mathbf {y}}'_h\widehat{\mathbf {y}}_h)^{-1}\text {.} \end{aligned}$$

At each iteration of **Step 1**, **2**, **3**, **4** and **5**, the discrepancy function (18) decreases or at least does not increase. The algorithm stops when the discrepancy function (18) decreases less than a positive arbitrary constant. It is now required that the vector $\mathbf {m}_{1h}$ gets filled by nonnegative elements, and, consequently, the algorithm based on (18), (19), (20), (21), (22) and (23) has to be modified to include nonnegative constraints on $\mathbf {m}_{1h}$. The solution can be found by the nonnegative least-squares algorithm (Lawson & Hanson, 1974, pp. 168–169), which is an active set algorithm. The *H* inequality constraints are active if the regression coefficients $\mathbf {m}_{1h}$ in the loss function (27) are negative (or zero) when unconstrained estimated, otherwise constraints are passive. The nonnegative solution of (18) with respect to $\mathbf {m}_{1h}$ will simply be the unconstrained least-squares solution using only the MVs corresponding to the passive set, imposing the regression coefficients of the active set to zero. A similar step is used to constrain $\widehat{\mathbf {c}}$ to be nonnegative.

## Simulation study

In this section, a simulation study was implemented in order to assess the classification of MVs and evaluate the reliability of the first-order factors. Moreover, 2ON-DFA was compared with respect to EFA followed by rotations. Each simulated random sample of $n > J$ multivariate observations $\mathbf {x}_i \; (i=1,\dots ,n)$ is generated according to $\mathbf {X}=\mathbf {X}_t+\mathbf {E}$, where $\mathbf {X}_t \sim N_J(\mathbf {0},\mathbf {R}_{\mathbf {x}})$ and $\mathbf {E} \sim N_J(\mathbf {0},a \mathbf {I}_J)$, where *a* allows to set an error. The correlation structure $\mathbf {R}_{\mathbf {x}}$ is modeled as follows:

30 $$\begin{aligned} \mathbf {R}_{\mathbf {x}}=\beta (\mathbf {VV}'-\mathbf {I}_J)+\zeta (\mathbf {1}_J\mathbf {1}'_J-\mathbf {VV}')+\mathbf {I}_J \end{aligned}$$

where $\beta =0.8+0.05\delta$, $\zeta =0.35+0.05\delta$, with $\delta \sim N(0,1)$ and $\beta >\zeta$. In detail, $\beta$ represents the correlation among MVs related to the same first-order factor (i.e., the elements in Eq. 15) and $\zeta$ represents the correlation among MVs relate to different first-order factors (i.e., the elements in Eq. 17).

In particular, the following scenarios were considered in the simulation study:

- number of MVs, $J=20,50$;
- number of the first-order factors, $H=5,10$;
- different error levels, $a=0.33,0.66,0.85,1,1.33$.

500 datasets containing 200 observations were generated for each setting and the data were standardized. In addition, a different random membership matrix $\mathbf {V}$, representing a random partition of *J* MVs in *H* nonempty subsets, was generated for each dataset. In detail, the matrix $\mathbf {V}=[\mathbf {v}_1,\dots ,\mathbf {v}_J]'$ was generated according to $\mathbf {v}_j \sim \text {Multinomial}(H: p_h=\frac{1}{H}, \; h=1,\dots ,H), \; \forall j=1,\dots ,H$. The algorithm was run from 30 random starting points to increment the possibility of obtaining the optimal solution, although it was observed that a few random starting points were generally enough. The models’ performance was analyzed using ARI (Hubert & Arabie, 1985), comparing the simple structure of the MVs generated by the true matrix $\mathbf {V}$ with the $\widehat{\mathbf {V}}$ estimated by the 2ON-DFA (with *H* first-order factors). In fact, ARI ranges between 0 and 1, where 1 implies that the estimated partition $\widehat{\mathbf {V}}$ is identical to the true $\mathbf {V}$.

We therefore counted the percentage of times that ARI is equal to 1 and the average of ARI in the 500 experiments. The model was also evaluated with Bayesian Information Criterion (BIC, Schwarz, 1978), Akaike’s Information Criterion (AIC, Akaike, 1973) and Root Mean Square of Residuals (RMSR, Hooper et al., 2008) for the correlation matrix.

**Fig. 3:**
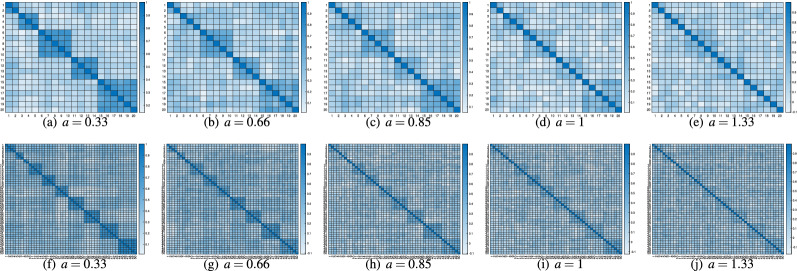
Heatmaps of examples of correlation matrix produced by the simulation study with different levels of error. First row: scenario $n=200$, $J=20$, $H=5$; second row: scenario $n=200$, $J=50$, $H=10$

In Fig. 3, we showed examples of generated correlation matrices with different level of errors in order to allow the reader to visually appreciate the meaning of low, medium and high errors. As we can see, in Fig. 3a, b, f, g the correlation matrices were generated with low error and the block diagonal structure is clearly visible, whereas in Fig. 3c, d, h, i the correlation matrices were generated with medium error and the block diagonal structure is less clear, but still visible. Finally, in Fig. 3e, j the structure is entirely invisible.

Tables 1 and 2 report the performances of the model values of the following indices: the average of ARI, BIC, AIC and RMSR on the 500 generated datasets and the percentage of ARI equal to 1. On average, in Table 1, ARI decreases from 1 to 0.85 when the error increases, and the model detects the real correlation structure most of the times for *a* equal to 0.33, 0.66, 0.85, 1. Inferior performances in terms of $\%$ of ARI equal to 1 for $a=1.33$ are recorded. Nevertheless, the performances of the model in scenario $n=200$, $J=20$, $H=5$ are appreciable even when the block diagonal structure tends to be less visible. RMSR always indicates a good fit of the model (i.e., values always lower than or equal to 0.08). In Table 2, ARI’s mean values are always greater than 0.78. Thus, the performances of the model in scenario $n=200$, $J=50$, $H=10$ are noticeable considering the average of ARI as figure of merit, even when the model identifies the true partition only in $20\%$ of all cases. RMSR never assumes a value higher than 0.09. Even if BIC and AIC, as expected, show a decreasing performance of 2O-DFA as the error increases, the slope of this decrement tends to reduce when the error increases. Additionally, BIC and AIC of the optimal solution of 2O-DFA ($\text {BIC}_\text {opt}$ and $\text {AIC}_\text {opt}$, respectively) were compared with BIC and AIC of a random block diagonal structure of model (6) ($\text {BIC}_\text {rand}$ and $\text {AIC}_\text {rand}$, respectively), i.e., with an initial random solution of model (6). This comparison was included because individual AIC and BIC values are not interpretable as they contain arbitrary constants and are much affected by sample size. A comparison was therefore imperative (Burnham & Anderson, 2002). Tables 1 and 2 show that $\text {BIC}_\text {rand}$ and $\text {AIC}_\text {rand}$ are approximately between 5.4 and 1.21 times worse than $\text {BIC}_\text {opt}$ and $\text {AIC}_\text {opt}$ in all scenarios, showing how the optimal solution of 2O-DFA is different from a random solution as the error increases.

**Table 1:** Simulated datasets with $n=200$, $J=20$, $H=5$ and different levels of error

Criterion	$a = 0.33$	$a = 0.66$	$a = 0.85$	$a = 1$	$a = 1.33$
ARI	1	1	0.99	0.97	0.85
$\%$ ARI	100	100	92	72	26
BIC	1131	2155	2402	2523	2660
AIC	1314	2038	2285	2406	2543
$\text {BIC}_\text {rand} / \text {BIC}_\text {opt}$	5.1	2.11	1.64	1.44	1.21
$\text {AIC}_\text {rand} / \text {AIC}_\text {opt}$	5.4	2.17	1.67	1.45	1.22
RMSR	0.02	0.03	0.04	0.05	0.08

*a* sets the level of error; rand and opt represent the solutions of a random block diagonal structure model and 2O-DFA, respectively.

**Table 2:** Simulated datasets with $n=200$, $J=50$, $H=10$ and different levels of error

Criterion	$a = 0.33$	$a = 0.66$	$a = 0.85$	$a = 1$	$a = 1.33$
ARI	1	0.99	0.98	0.96	0.78
$\%$ ARI	100	84	55	38	20
BIC	7140	8990	9621	10010	10327
AIC	6854	8703	9334	9723	10040
$\text {BIC}_\text {rand} / \text {BIC}_\text {opt}$	4.6	2.22	1.70	1.49	1.26
$\text {AIC}_\text {rand} / \text {AIC}_\text {opt}$	4.7	2.27	1.73	1.50	1.27
RMSR	0.02	0.04	0.04	0.06	0.09

*a* sets the level of error; rand and opt represent the solutions of a random block diagonal structure model and 2O-DFA, respectively.

The simulation study additionally examined the ability of the model to build reliable first-order factors via Cronbach’s $\alpha$ (Cronbach, 1951), which can be seen as the expected correlation between all pairs of MVs used to specify the concept (Chaouachi & Rached, 2012; Fornell & Larcker, 1981; Kline, 2000). Cronbach’s $\alpha$ ranges between negative infinity and 1. Tables 3 and 4 show evaluations of internal consistency of the first-order factors estimated by the model.

**Table 3:** Percentage of times Cronbach’s alpha (computed for each subset of MVs) $> 0.9$ with different scenario and different levels of error

Scenario	$a = 0.33$	$a = 0.66$	$a = 0.85$	$a = 1$	$a = 1.33$
$n=200$, $J=20$, $H=5$	90.5	70.9	14.4	7.3	6.8
$n=200$, $J=50$, $H=10$	85.3	84.2	33	28.2	21.7

**Table 4:** Percentage of times Cronbach’s alpha (computed for each subset of MVs) $< 0.7$ with different scenario and different levels of error

Scenario	$a = 0.33$	$a = 0.66$	$a = 0.85$	$a = 1$	$a = 1.33$
$n=200$, $J=20$, $H=5$	0	0	9.2	11.1	14.2
$n=200$, $J=50$, $H=10$	0.4	0.5	6.2	8.3	10.8

The results in Table 3 could lead the reader to think that the proposed model does not provide reliable first-order factors when *a* is equal to 1 and 1.33; but looking at Table 4, we can state that Cronbach’s $\alpha$, computed for each subset of MVs, is always higher than 0.70 in settings with $a=0.33$ and $a=0.66$ for the scenario $n=200$, $J=20$, $H=5$ and almost always for the scenario $n=200$, $J=50$, $H=10$. Overall, most of the time the method is able to get reliable first-order factors, at least $85.8\%$.

We also conducted an additional study to assess the performance of the 2ON-DFA in detecting a SSM, with respect to EFA followed by rotation, where each variable was assigned to the factor with the highest loading (in absolute value) resulting from EFA followed by a rotation method. For the rotation, we considered the four widely used methods: Varimax, Promax, Quartimin and Geomin (Abdi, 2003; Browne, 2001). Moreover, 2O-DFA’s performance was compared with the results obtained by SSFA (Adachi & Trendafilov, 2018) and FANC (Hirose & Yamamoto, 2014). SSFA was performed imposing the number of common factors equal to *H*, a convergence tolerance equal to $10^5$ and 50 different initial solutions for the loading matrix. Let us recall that FANC is formulated as follows:

31 $$\begin{aligned} \min _{\mathbf {A},{\varvec{\Sigma }}_{\mathbf {y}},{\varvec{\Psi }}_{\mathbf {x}}} l(\mathbf {A},{\varvec{\Sigma }}_{\mathbf {y}},{\varvec{\Psi }}_{\mathbf {x}}) + \rho P_{\gamma }(\mathbf {A}) \end{aligned}$$

where $P_{\gamma }(\mathbf {A})$ penalizes $\mathbf {A}$ to have nonzero elements, with $\gamma$ and $\rho$ being tuning parameters. Since finding the tuning parameters that provide sparsest loading matrices was exceedingly onerous, FANC was performed imposing $\gamma =1.01$ and with $\rho$ selected according to BIC in order to have a SSM to compare with the other methods.

The parameters considered in this Section were reused also for this comparison: 500 datasets, $n=200$, $J=20,50$, $H=5,10$ and $a=0.33,0.66,0.85,1,1.33$. In Table 5, it is possible to see that 2ON-DFA outperforms the other methods considering both the average of ARI between the generated partition of variables and the partition predicted by the methodologies and the percentage of ARI equal to 1 as figures of merit.

**Table 5:** Comparison among methods to detect the SSM on 500 datasets

Methods	$a = 0.33$	$a = 0.66$	$a = 0.85$	$a = 1$	$a = 1.33$
Scenario $n=200$, $J=20$, $H=5$					
Second-Order DFA	1 (100)	1 (100)	0.99 (95)	0.96 (69)	0.88 (31)
EFA + Varimax	1 (100)	0.99 (98)	0.95 (73)	0.91 (51)	0.69 (10)
EFA + Promax	1 (100)	0.99 (98)	0.95 (75)	0.90 (48)	0.67 (8)
EFA + Quartimin	1 (100)	0.99 (98)	0.95 (77)	0.92 (53)	0.69 (11)
EFA + Geomin	1 (100)	0.99 (98)	0.95 (76)	0.91 (53)	0.70 (10)
SSFA	1 (100)	0.95 (59)	0.76 (5)	0.59 (0)	0.35 (0)
FANC	1 (100)	1 (100)	0.96 (75)	0.92 (50)	0.85 (28)
Scenario $n=200$, $J=50$, $H=10$					
Second-Order DFA	1 (100)	0.99 (89)	0.99 (61)	0.95 (32)	0.79 (25)
EFA + Varimax	0.99 (99)	0.98 (65)	0.95 (24)	0.89 (3)	0.64 (0)
EFA + Promax	0.99 (99)	0.98 (70)	0.95 (29)	0.88 (4)	0.61 (0)
EFA + Quartimin	0.99 (99)	0.98 (72)	0.97 (53)	0.89 (21)	0.65 (6)
EFA + Geomin	0.99 (99)	0.99 (73)	0.97 (54)	0.90 (23)	0.66 (6)
SSFA	0.43 (0)	0.40 (0)	0.39 (0)	0.33 (0)	0.18 (0)
FANC	0.22 (0)	0.22 (0)	0.21 (0)	0.21 (0)	0.18 (0)

The results indicates the average of ARI ($\%$ of ARI equal to 1).

It is evident that our proposal is able to detect the SSM better than both the sequential application of EFA followed by rotation, SSFA and FANC, while also simultaneously estimating the correlation between the first-order factors.

## Application

Our proposal was applied on the OECD data on *Better Life Index 2015*,^1^ an initiative started in 2011 which focused on statistics that capture important aspects of life and that characterize the quality of people’s lives. The dataset is formed by 34 countries and 24 variables which reflect two pillars that OECD identified as essential to well-being:

*Material Living Conditions* (MLC) is specified by three dimensions (characterized by their own sub-dimensions): *Housing* (*Dwellings without basic facilities*, *Housing expenditure* and *Rooms per person*), *Income* (*Household net adjusted disposable income* and *Household net financial wealth*) and *Jobs* (*Employment rate*, *Job security*, *Long-term unemployment rate* and *Personal earnings*).

*Quality of Life* (QL) is identified by eight dimensions (characterized by their own sub-dimensions): *Community* (*Quality of social support network*), *Education* (*Educational attainment*, *Student skills* and *Years in education*), *Environment* (*Air pollution* and *Water quality*), *Civic Engagement* (*Consultation on rule-making* and *Voter turnout*), *Health* (*Life expectancy* and *Self-reported health*), *Life Satisfaction* (*Life Satisfaction*), *Safety* (*Assault rate* and *Homicide rate*) and *Work-Life Balance* (*Employees working very long hours* and *Time devoted to leisure and personal care*).

2ON-DFA is scale invariant, thus the model results equivariant under an affine transformation of the data. In this application, MVs were standardized, yet the re-scaling normalization (also known as Min-Max normalization, which converts MVs to the same scale and makes all the values fall within the interval [0, 1]) could also be used obtaining exactly the same results.

Since 2ON-DFA identified a system of nonnegative loadings, the first application was used to detect eventual MVs that could measure a negative component of the general latent construct, in our case the well-being. The number of specific constructs *H* was fixed equal to the minimum 2. When MVs had positive loadings, it meant that they were positive dimensions of the general latent construct.

However, if *H* is optimally chosen and there still is a MV with no significant loading, this suggests discarding the MV from the analysis because it is irrelevant within the model; the MV can actually be confounding for the analysis and it could be removed without incurring into a significant loss of information. Finally, if some MVs have exactly zero loadings on the two factors of the model, this means that they measure a negative component of the general latent construct and therefore that they must be reversed.

In our analysis, MVs (1), (2), (7), (8), (14), (21), (22) and (23) loaded zero on the two factors (i.e., a row with zero loadings for each of the above MVs was observed). In fact, these MVs measured a negative component of well-being and therefore had no positive loadings to show; thus, they were reversed by changing the sign (i.e., by multiplying each MV by $-1$). When reapplying the model with $H=2$, all MVs loaded on factors with a positive loading except for MVs (2) (*Housing expenditure*) and (16) (*Consultation on rule-making*). Under the hypothesis $H=2$, the MVs (2) and (16) are, therefore, irrelevant in the model and were consequently removed. The subsequent analyses were repeated without these two MVs.

In the first application of the model, the 22 MVs were constrained to define the MLC and QL factors as indicated by the OECD. The results of the analysis are reported in Table 6 columns 1 and 2. The methodology resulted in a second-order nonnegative disjoint confirmative factor analysis because all MVs were forced a priori to load on the specified MLC and QL as indicated by OECD, and all loadings were hence constrained to be nonnegative. BIC and AIC resulted 661.76 and 591.55, respectively. The two identified factors were not unidimensional constructs, since the second largest eigenvalue of the variance–covariance sub-matrices related to the two subsets of MVs were clearly larger than 1 and equal to 1.88 and 2.16. However, the reliability or internal consistency of each factor measured by Cronbach’s $\alpha$ was good (Kline, 2000) and equal to 0.87 for both subsets. The internal consistency is the extent to which MVs, associated with a factor, measure a unique theoretical construct. The observed results suggested verifying if the selected model could be statistically confirmed by the data of 2015. A common approach for model selection is to use theory to specify an initial model, in our case the MLC and QL dimensions defined by OECD, and then to use the likelihood ratio $\chi ^2_\nu$ test to decide whether the model is confirmed or should be simplified or expanded (Bollen, 1989). However, this test is problematic in practice due to its sensitivity to the sample size and to its tendency to reject good models. This situation is well-known in literature and for this reason Marsh, Balla and McDonald (1988) and several other authors proposed more than 30 alternative measures. Among these, we considered the two information criteria BIC and AIC that proved to work well on SEM and confirmatory factor analysis problems (Raftery, 1995).

**Table 6:** Analysis of different second-order factor analysis models for defining two dimensions of wellbeing: material living conditions and quality of life

Column		1	2	3	4	5	6
		OECD		Constrained		Unconstrained	
		MLC	QL	MLC	QL	MLC	QL
*First-order factors*							
1. Housing	1	0.61			0.62	0.64	
	2						
	3	0.80		$\mathbf{0}.81$		0.82	
2. Income	4	0.95		$\mathbf{0}.95$		0.92	
	5	0.75		0.74		0.71	
3. Jobs	6	0.63			0.85		0.85
	7	0.27			0.42		0.45
	8	0.33			0.39		0.41
	9	0.94		$\mathbf{0}.94$		0.96	
4. Community	10		0.69		$\mathbf{0}.60$	0.56	
5. Education	11		0.62		0.58		0.54
	12		0.68		$\mathbf{0}.55$	0.44	
	13		0.62		0.53		0.54
6. Environment	14		0.56		0.52		0.48
	15		0.78		$\mathbf{0}.95$		0.99
7. Civic engagement	16						
	17		0.21	0.43		0.43	
8. Health	18		0.59		$\mathbf{0}.55$	0.70	
	19		0.29	0.52		0.53	
9. Life satisfaction	20		0.44		$\mathbf {0.62}$	0.71	
10. Safety	21		0.63	0.38		0.39	
	22		0.67		$\mathbf {0.42}$		0.38
11. Work-life balance	23		0.67		$\mathbf{0}.55$		0.51
	24		0.54		0.40	0.37	
*Second-order factor*		0.88	0.89	0.91	0.88	0.88	0.88
Communality		3.95	4.87	3.59	5.28	5.61	3.29
Cronbach’s $\alpha$		0.87	0.87	0.88	0.85	0.90	0.82
Unidimensionality		1.87	2.16	2.88	1.13	2.24	1.80
BIC		661.76		642.22		630.73	
AIC		591.55		572.01		560.52	
Discrepancy		192.50		140.54		134.16	
Total communality		10.40		10.42		10.46	

Constrained MVs are reported in bold in columns 3 and 4.

In order to examine how much the latent constructs MLC and QL change by considering a less constrained model, the 2ON-DFA was repeated by forcing only a single MV for each of the 11 dimensions to load on MLC or QL as indicated by OECD. In practice, the MV that in the first analysis had the maximal loading within its dimension was forced to load as indicated by OECD on MLC or QL. The dimension *Civic engagement* (7) was characterized only by the MV *Voter turnout* which had a low loading (0.21) in the OECD proposal. This MV was thus left free to load on one of the two latent constructs, either MLC or QL. The 10 constrained MVs are reported in bold in columns 3 and 4 of Table 6. The construct MLC has: *Housing* represented by *Rooms per person* (3); *Income by Household net adjusted disposable income* (4); and *Jobs* by *Personal earnings* (9). The construct QL has: *Community* represented by *Quality of support network* (10); *Education* by *Student skills* (12); *Environment* by *Water quality* (15); *Health* by *Life expectancy* (18); *Life Satisfaction* by *Life Satisfaction* (20); *Safety* by *Homicide rate* (22); *Work-Life Balance* by *Employees working very long hours* (23). The rest of MVs was left free to load on one of the two latent constructs.

After constraining 10 out of the 22 MVs to define MLC and QL as indicated by OECD, it was expected that the new analysis confirmed that also the rest of the MVs followed the two hypothesized latent constructs. This proved false for 7 of the remaining 11 MVs. In Table 6, columns 3 and 4 show the optimal solution. In this second analysis, the values of BIC and AIC were considerably lower, and equal to 642.22 and 572.01, respectively. According to the study of Raftery (1995), there is a “very strong” evidence that this model is better than the one proposed by OECD, since there is a difference in BIC between models larger than 10 ($\hbox {BIC}_\text {OECD} - \hbox {BIC}_\text {Con} = 20.54$). In this new application of our model, MVs *Employment rate*, *Job security* (reversed), *Long-term unemployment rate* (reversed) showed to be better related to the *Quality of Life*, while *Voter turnout*, *Self-reported health *and *Assault rate* (reversed) were better related to *Material Living Conditions*. Therefore, the *Quality of Life* defined by this constrained model also included the need for a secure and a long-term job, while the *Material Living Conditions* included the need for a good personal health condition, personal safety and interest to become involved in the political process.

It remained to understand if these two latent constructs defined could be further improved by leaving all the MVs free to load on one of the two latent constructs. 2ON-DFA was therefore applied for a third time, yet without constraints on the MVs. The best solution over 100 runs was retained and the results were reported in Table 6, columns 5 and 6. Although the values of BIC and AIC decreased to 630.73 and 560.52, the reduction was not as strong as in the second application of our proposal. However, the difference of BIC ($\hbox {BIC}_\text {Con} - \hbox {BIC}_\text {Unc} = 12.65$) was still extremely relevant. Differently from the OECD model, *Life Satisfaction*, *Community* (*Quality of support network*), *Students Skills* and *Time devoted to leisure and personal care* loaded on MLC in the unconstrained model. *Life expectancy*—the other MV of the *Health* dimension—loaded on MLC. Therefore, the MLC construct of the unconstrained model also included *Life Satisfaction*, *Community* and part of *Work-Life Balance*. It is worth observing that the subsets of specific MVs proposed by OECD to define the 11 dimensions were frequently “incoherent”, in the sense that they loaded on different latent constructs. For example, the two MVs *Employees working very long hours* and *Time devoted to leisure and personal care* that defined the dimension *Work-Life Balance*, actually loaded on MLC and QL, respectively. The same happens for *Safety*, *Education* and *Jobs*. This thereby suggested that the proposed MVs, when considered all together, were not coherent with the hypothesized theoretical construct.

### The optimal number of dimensions for well-being

The analysis was repeated for different values of *H* in order to find the solution with the lowest BIC and AIC and to understand if there were more than two pillar dimensions (MLC and QL). For $H=5$, BIC and AIC resulted as the lowest, with 590.07 and 515.27, respectively. Note that MVs (2) and (16) were re-inserted in the analysis; however, these increased drastically BIC and AIC and were discarded again.

The five factors were almost all unidimensional since the second largest eigenvalue of the variance–covariance sub-matrices related to the five subsets was not strongly larger than 1 and, in four subsets, it was actually lower (1.024, 0.619, 0.770, 0.795, 0.261). Cronbach’s $\alpha$ was equal to 0.88, 0.86, 0.82, 0.77, 0.85, respectively, thus confirming the good internal consistency of factors. The results are reported in Table 7.

**Table 7:** Analysis of second-order factor analysis model for defining five dimensions of well-being

Column		1	2	3	4	5
		MLC	ES	QS	QSHH	J
*First-order factors*						
1. Housing	1				0.94	
	2					
	3	0.81				
2. Income	4	0.94				
	5	0.72				
3. Jobs	6			0.84		
	7					0.86
	8					0.86
	9	0.97				
4. Community	10			0.56		
5. Education	11		0.66			
	12		0.76			
	13			0.55		
6. Environment	14				0.58	
	15			0.99		
7. Civic engagement	16					
	17	0.44				
8. Health	18	0.70				
	19				0.49	
9. Life satisfaction	20	0.71				
10. Safety	21		0.92			
	22		0.76			
11. Work-life balance	23				0.70	
	24	0.35				
*Second-order factor*		0.92	0.35	0.65	0.82	0.77
Communality		4.26	2.44	2.32	1.95	1.48
Cronbach’s $\alpha$		0.88	0.86	0.82	0.77	0.85
Unidimensionality		1.02	0.62	0.77	0.80	0.26
BIC				590.07		
AIC				515.27		
Discrepancy				104.17		
Total communality				15.11		

The first latent construct was mostly MLC including *Civic Engagement* (*Voter turnout*), *Health* (*Life expectancy*), *Life Satisfaction* and *Work-Life Balance*. This factor was mainly formed by a subset of MVs defining MLC in the unconstrained solution of the 2-factor model. It is interesting to observe that there was another factor for MLC, the fifth, dedicated to the dimension *Jobs* with *Job insecurity* (reversed) and *Long-term unemployment rate* (reversed). The other three factors eventually split the QL into first-order factors. The second factor was formed by *Education* (*Educational attainment*, *Student skills*) and *Safety* (*Assault rate*, *Homicide rate* both reversed) and therefore measured the level of safety and education in *Society* as a relevant aspect of the QL. The third factor measured the *Quality of Society* in terms of *Community* (*Quality of social support network*), *Environment* (*Water quality*), *Years in education* and *Employment rate*. Therefore, the latter measured fundamental aspects for *Society* such as the quality of the social network support, the quality of the water, the quality of education and the quality of working life. The fourth factor measured the *Quality of Habitat for Humanity* based on *Dwellings without basic facilities* (reversed), *Air pollution* (reversed), *Self-reported health* and *Employees working very long hours* (reversed). The fourth factor, thus, focused on the quality of the habitat for the humanity based on decent and affordable housing, good air quality, the perceived health in the habitat and the working stability of the habitat.

## Conclusion

Our proposal, *Second-Order Disjoint Factor Analysis*, allows modeling an unknown hierarchical structure of the MVs with two orders. It is a second-order factor analysis with a disjoint structure for the MVs. Each subset of MVs is detected to be reliable, that is, MVs related to a first-order factor consistently measure a unique theoretical construct. The second-order represents the general factor which summarizes the common information related to the *H* specific first-order factors. A nonnegative version of the model is presented which entails that MVs are concordant with the related factor and, therefore, the associated factor loadings are constrained to be nonnegative. According to the distributional assumptions, the maximum likelihood estimation of the models allows us to make inference on the parameters. Since the maximization of the likelihood represents a discrete and continuous problem that cannot be solved by a quasi-Newton type algorithm, the estimation of all parameters is simultaneously obtained following a cyclic block coordinate descendent algorithm. An application about well-being presents the characteristics of the new methodology, which shows in detail the limitation of the confirmatory analysis when the framework proposed is not respected by the data. Four models are proposed: a fully confirmatory analysis (framework and number of factors are given), a semi-confirmatory analysis (main relations and number of factors are given), an exploratory analysis (only the number of factors is given) and a full exploratory analysis (the number of factors is selected by an information criterion). This latter shows the real potential of our proposal: the detection of the best partition of MVs, and thus of the latent concepts and the related factors; and, moreover, their aggregation into a second-order factor.

The paper, thus, provides a generalization of both hierarchical factor analysis and non-orthogonal disjoint factor analysis, and proposes a constrained version with the additional request of nonnegativity of loadings.

Nevertheless, some further challenges are foreseen within our research. Our future goal is to develop the inclusion of cross-loadings in the SSM in order to increase the fit of the model using the work by Vichi (2017). Indeed, by applying 2O-DFA it may occur that sample covariance matrix is block diagonal, but that there are relevant cross-loadings between blocks, i.e., some variables belonging to different blocks are highly correlated. Here, the 2O-DFA should include the estimation of cross-loadings and therefore define factors that are not disjoint. Moreover, another important development consists of the introduction of a time dependence in order to provide a model able to fit the phenomenon of study throughout the years. Two ideas are currently being developed: the first is that of encompassing a time dependence in the loading matrices, as already done by Maruotti and Vichi (2014) on the centroids matrices in their time-varying extension of *k*-means. The second idea consists of the inclusion of a hidden Markov model (HMM) in order to follow the evolution of MVs depending on both the first-order factors and on the general one (i.e., the second-order factor).

## Footnotes

1 Available at https://stats.oecd.org/Index.aspx?DataSetCode=BLI2015.
